# Supplementary material for: Identification of Freezing-Responsive microRNAs and Their Targets in Chinese Jujube by Small RNA and Degradome Sequencing
Source: Int J Mol Sci. 2026 Sep 4;27(17):7912. doi: 10.3390/ijms27177912 (PMC13566890; doi:10.3390/ijms27177912)
Supplement: Supplementary file 1 [file ijms-27-07912-s001.zip › Supplementary Material S2/novel_miR_73.pdf]

Provisional ID	:	novel_miR_73
Score total	:	0.4
Score for star read(s)	:	-1.3
Score for read counts	:	0
Score for mfe	:	0.1
Score for randfold	:	1.6
Score for cons. seed	:	
Total read count	:	201
Mature read count	:	201
Loop read count	:	0
Star read count	:	0

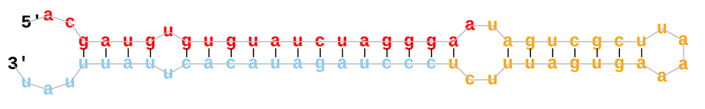

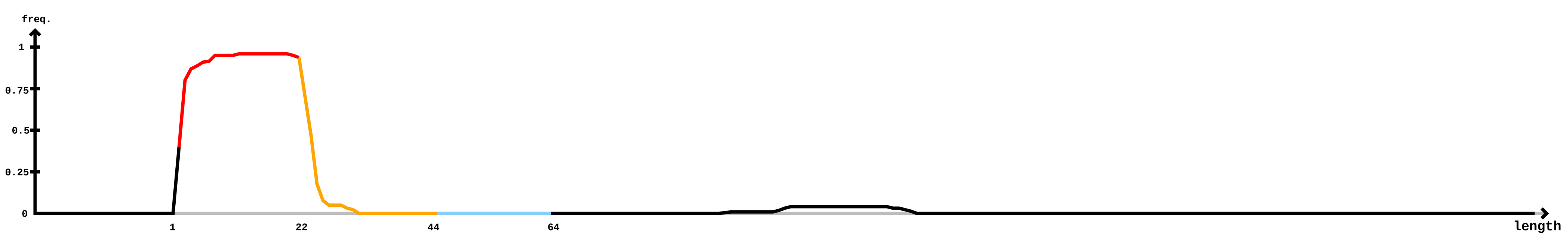

Mature Star

5'- aauugugaaauaaauaaaaaauacgauguguguaucuagggaauagucgcuuaaaagugauuucucccuagauacacuuauuuauucaauucaauuuuggaucuauucagacuauauggaaggugcuaaagauucaaaacucauuuuuauccuuuuuuuuuaaauaaaaaaaauaaauccauuaaaauuuaugcaaaaugcuuuucuauuucuauaauguccaauauauguuuuauauauucuaugcgaa -3' exp

.((((((((((......((((.((((.(((((((((((((..(((((((....)))))))..))))))))))))).))))))))........((((((((((.....(((........)))......))))))))))............((((((......))))))((((((........))))))(((...))).....)))))).)))))....((((((((...))))))))..........	reads	mm	sample
.....................acgauguguguaucuagggaa...	46	0	LCK-2
.....................acgauguguguaucuagggaa...	35	0	LCK-1
.....................acgauguguguaucuagggaC...	1	1	LT-1
.....................acgauguguguaucuagggaau...	3	0	LCK-3
.....................acgauguguguaucuagggaau...	1	0	LT-3
.....................acgauguguguaucuagggaau...	1	0	LT-1
.....................acgauguguguaucuagggaaC...	1	1	LT-3
......................cgauguguguaucuaggg...	1	0	JCK-1
......................cgauguguguaucuaggg...	1	0	JCK-2
......................cgauguguguaucuaggga...	2	0	LT-1
......................cgauguguguaucuaggga...	1	0	LT-3
......................cgauguguguaucuagggaaA...	1	1	LT-3
......................cgauguguguaucuagggaau...	3	0	LT-3
......................cgauguguguaucuagggaau...	1	0	JCK-3
......................cgauguguguaucuagggaaC...	1	1	LT-3
......................cgauguguguaucuagggaau...	3	0	JCK-2
......................cgauguguguaucuagggaau...	2	0	LT-1
......................cgauguguguaucuagggaaua...	24	0	LCK-2
......................cgauguguguaucuagggaaua...	29	0	LCK-1
......................cgauguguguaucuagggaaua...	2	0	JT-3
......................cgauguguguaucuagggaaua...	2	0	JT-2
......................cgauguguguaucuagggaauag...	13	0	LCK-3
......................cgauguguguaucuagggaauagucgcuu...	1	0	JCK-1
......................cgauguguguaucuagggaauagucgcuu...	2	0	JCK-3
.......................gauguguguaucuagggaau...	1	0	LT-1
.......................gauguguguaucuagggaau...	1	0	LT-3
.......................gauguguguaucuagggaaC...	1	1	LT-3
.......................gauguguguaucuagggaauC...	6	1	LCK-3
.......................gauguguguaucuagggaauagA...	6	1	LCK-3
........................auguguguaucuagggaa...	1	0	LT-3
........................auguguguaucuagggaauagucgc...	1	0	JCK-1
........................auUuguguaucuagggaauagucgc...	1	1	JCK-1
........................auguguguaucuagggaauagucgc...	1	0	JCK-2
.........................uguguguaucuagggaaua...	1	0	JCK-3

```
                    Mature                              Star
aauugugaaauaaauaaaaaauacgauguguguaucuagggaauagucgcuuaaaagugauuucucccuagauacacuuauuuauucaauucaauuuuggaucuauucagacuauauggaaggugcuaaagauucaaaacucauuuuuauccuuuuuuuuuaaauaaaaaaaauaaauccauuaaaauuuaugcaaaaugcuuuucuauuucuauaaauguccaauauauguuuuauauauucuaugcgaa
...ugugguaucuagggaauag...                      1   0   JCK-1
...ugugguaucuagggaauagucgcu...                 1   0   LT-2
...ugugguaucuagggaauagucgcu...                 1   0   LT-1
...ugugguaucuagggaauagucgcuu...                1   0   JCK-2
...guguguaucuagggaaua...                       1   0   LT-3
...uguguaucuagggaauag...                       3   0   JT-2
...uguguaucuagggaauag...                       5   0   JT-3
...uaucuagggaauagucgc...                       1   0   LT-3
...uaucuagggaauagucgcuu...                     1   0   LT-3
...auauggaaUgugcuaaagauucaaaacuca...           1   1   JCK-3
...uauggaaUgugcuaaagauucaaaacuca...            1   1   LT-1
...gugcuaaagauucaaaacu...                      2   0   LCK-3
...ugcuaaagauucaaaacucau...                    2   0   LT-1
...ugcuaaagauucaaaacucauu...                   1   0   LT-3
...gcuaaagauucaaaacucauu...                    1   0   LT-3
...gcuaaagauucaaaacucauu...                    1   0   LT-2
```
